# Reconceptualising the DSM: Neuroanalysis and digital brain profiling

**DOI:** 10.1192/j.eurpsy.2021.70

**Published:** 2021-08-13

**Authors:** A. Peled

**Affiliations:** Dept' Sm, Mental Health Center, ‘Technion’ Israel Institute of Technology, Haifa, Israel

**Keywords:** psychiatry, diagnosis, digital, Brain

## Abstract

Recent years have seen a great advancement in the emerging field of Neural Computation, a study of the brain using neuronal network models. As a consequence, another field of science is being developed titled ‘Computational Psychiatry’ where neuronal network models of psychopathology help understand the possible etiology for mental disorders. With Computational Psychiatry we can begin and reformulate mental disorders as brain disorders. Etiological diagnosis in psychiatry will be the next breakthrough which will allow to effectively treat mental disorders and will bring psychiatry back to the realm of medicine Computational Psychiatry together with advances in technology, will transform psychiatry beyond recognition: With the development of the connecting internet and sensor technology (e.g., face speech recognition) mental status examination can be easily extracted and delivered over distance (tele-psychiatry). With the help of AI the extracted psychiatric phenomenology can be interpreted to match most of the diagnostic process of a skilled psychiatrist. Once achieved a continual psychiatric monitoring coupled with new technology of wireless dry-electrode electrophysiological brain imaging can begin and collect big-data. Big-data analysis stand a good chance to reveal the etiological correlations between mental disorders and their brain-related origins. Thus, etiology for mental disorders can begin to unravel. Neural modulation technology will be the answer for effective therapeutic interventions (i.e., future brain pacers).

**Disclosure:**

I am in a preliminary effort to develop a Digital application in the field of Psychiatry

